# Comparative green synthesis and physicochemical characterization of silver nanoparticles using *Thymus vulgaris* extracts from Syrian and Yemeni sources

**DOI:** 10.7717/peerj.21583

**Published:** 2026-08-04

**Authors:** Aiman Abdullah Ammari, Ramzi Ahmed Amran, Bandar Mohsen ALmunqedhi, Ahmad Rashed Alhimaidi

**Affiliations:** 1 Department of Zoology, College of Science, King Saud University, Riyadh, Saudi Arabia; 2 Department OF Botany and Microbiology, College of Science, King Saud University, Riyadh, Saudi Arabia

**Keywords:** Silver nanoparticles, Green synthesis, *Thymus vulgaris*, Nanoparticle characterization, Plant-mediated synthesis

## Abstract

Environmentally benign synthesis of silver nanoparticles (AgNPs) using plant-derived materials has emerged as an attractive strategy for producing functional nanomaterials while avoiding hazardous chemical procedures. In the present study, aqueous extracts of *Thymus vulgaris* obtained from Syrian and Yemeni commercial sources were employed for the green synthesis of AgNPs, followed by a comparative physicochemical characterization. To our knowledge, this is among the few studies comparing the physicochemical characteristics of AgNPs synthesized using *T. vulgaris* extracts obtained from two different commercial sources. UV-Vis spectroscopy revealed distinct absorption bands at 319.1 nm and 282.9 nm for the Syrian- and Yemeni-derived preparations, respectively. Dynamic light scattering analysis showed hydrodynamic diameters of 84.18 nm and 125.2 nm, whereas zeta potential measurements yielded values of −26.9 mV and −30.1 mV, indicating negatively charged nanoparticle surfaces. Quantitative TEM analysis based on measurements of 50 nanoparticles per sample revealed average particle diameters of 13.38 ± 5.26 nm and 12.34 ± 5.31 nm for Syrian- and Yemeni-derived AgNPs, respectively. SEM and TEM analyses demonstrated predominantly spherical nanoparticles with varying degrees of aggregation. EDS analysis confirmed silver as the major elemental constituent, while XRD analysis verified the crystalline metallic nature of the nanoparticles with a face-centered cubic structure and estimated crystallite sizes of 11.3 nm and 8.3 nm, respectively. Overall, the findings demonstrate that *Thymus vulgaris* extracts can serve as effective reducing and stabilizing agents for the green synthesis of crystalline silver nanoparticles. Although minor physicochemical differences were observed between the two nanoparticle preparations, further studies incorporating detailed phytochemical characterization are required to clarify the factors underlying these variations.

## Introduction

Nanotechnology has become a rapidly advancing field across materials science, medicine, and biotechnology due to the distinctive physicochemical characteristics of nanoparticles compared to bulk materials. Among metallic nanomaterials, silver nanoparticles (AgNPs) have attracted considerable interest owing to their notable antimicrobial properties, catalytic performance, optical behavior, and biocompatibility, which support their use in biomedical, diagnostic, and environmental applications (Khalifa et al., 2025; Bruna et al., 2021). Traditional approaches for AgNP synthesis, including chemical and physical methods, typically involve toxic reagents, elevated temperatures, and high energy consumption, highlighting the need for more sustainable strategies (Aboyewa et al., 2021).

Plant-mediated green synthesis has emerged as an effective and eco-friendly alternative for nanoparticle production. Bioactive compounds present in plant extracts—such as polyphenols, flavonoids, terpenoids, and reducing sugars—play dual roles as reducing and stabilizing agents, facilitating controlled nanoparticle formation without the use of hazardous chemicals (Thatyana et al., 2023; Bao et al., 2021). The composition of these phytochemicals is a key factor influencing nanoparticle size, morphology, stability, and functional properties (Wahab et al., 2021; Dhir et al., 2023; Thomas et al., 2024).

Recent studies have highlighted that the physicochemical characteristics of biosynthesized metallic nanoparticles are influenced not only by synthesis conditions but also by the composition and abundance of plant-derived metabolites involved in reduction and stabilization processes (Thomas et al., 2024; Lithi et al., 2025). Variations in plant genotype, environmental conditions, geographical location, soil composition, temperature, water availability, and cultivation practices may affect phytochemical profiles and consequently influence nanoparticle formation, size distribution, surface charge, and colloidal behavior (Sarfaraz, Rahimmalek & Saeidi, 2021; Etri & Pluhár, 2024; Zinno et al., 2023). Furthermore, recent investigations have emphasized that source-dependent variations in plant extracts may contribute to differences in the physicochemical characteristics of green-synthesized metallic nanoparticles, highlighting the importance of comparative studies involving plant materials obtained from different geographical origins (Şimşek, 2025; Rigopoulos et al., 2024).

*Thymus vulgaris* (thyme) is a well-known medicinal plant rich in phenolics and essential oils such as thymol, carvacrol, rosmarinic acid, and flavonoids—compounds that exhibit strong reducing and capping capabilities in nanoparticle biosynthesis (Ilić et al., 2022). Previous research has shown that T. vulgaris extracts can effectively mediate the formation of AgNPs with potent antimicrobial and antioxidant activity (Balciunaitiene et al., 2021; Aldosary et al., 2021). However, the influence of plant source on the physicochemical characteristics of thyme-mediated AgNPs remains insufficiently investigated.

Geographical variability between plant populations—including thyme materials originating from Syria and Yemen—may contribute to differences in secondary metabolite composition due to variations in environmental conditions, soil mineral content, altitude, temperature, and cultivation practices (Etri & Pluhár, 2024; Zinno et al., 2023). Evaluating whether such source-related variation is reflected in the physicochemical characteristics of biosynthesized nanoparticles may improve understanding of plant-mediated nanoparticle synthesis and support efforts toward improved reproducibility and optimization of green nanomaterial production.

Accordingly, this investigation explored the biosynthesis of silver nanoparticles (AgNPs) employing aqueous extracts of *Thymus vulgaris* obtained from two different commercial sources originating from Syria and Yemen. The produced nanoparticles were subjected to comparative physicochemical evaluation using a range of analytical methods, including UV–visible spectroscopy, dynamic light scattering (DLS), zeta potential measurements, scanning electron microscopy (SEM), transmission electron microscopy (TEM), energy-dispersive X-ray spectroscopy (EDS), and X-ray diffraction (XRD).

In addition, the study was designed to assess how geographical variation in plant origin may affect nanoparticle formation, dispersion behavior, surface stability, and structural organization. The obtained findings may contribute to improving the understanding of plant-mediated nanoparticle synthesis and support future applications of biosynthesized nanomaterials in biomedical and technological fields.

## Materials & Methods

### Plant material and authentication

Voucher specimens (accession numbers 24737 for the Syrian sample and 24736 for the Yemeni sample) were deposited in the Botanical Herbarium, College of Science, King Saud University. The herbarium does not currently maintain a publicly accessible online database for individual accession numbers. Dried aerial parts of *Thymus vulgaris* reportedly originating from Syria and Yemen were purchased from a local herbal market in Riyadh, Saudi Arabia. The plant materials were taxonomically authenticated at the Herbarium and Classification Unit, Department of Botany and Microbiology, College of Science, King Saud University, Riyadh, Saudi Arabia. Voucher specimens were deposited under accession numbers 24737 (Syrian sample) and 24736 (Yemeni sample). After authentication, the samples were washed, shade-dried at room temperature, and ground into a fine powder prior to extraction.

### Preparation of aqueous plant extracts

Preparation of the aqueous plant extracts was carried out according to widely adopted extraction approaches used in plant-mediated nanoparticle biosynthesis studies (Ahmed et al., 2016; Aboyewa et al., 2021). In brief, 10 g of finely powdered plant material was suspended in 100 mL of distilled water and maintained at 60 °C for 30 min under constant agitation to enhance the release of water-soluble phytochemicals. After completion of the extraction process, the suspension was allowed to stand until reaching ambient temperature and was then clarified through filtration using Whatman No. 1 filter paper. The resulting aqueous extract was stored at 4 °C and utilized in subsequent experimental procedures.

### Green synthesis of silver nanoparticles

Biosynthesis of silver nanoparticles was achieved using a plant-assisted reduction approach. For nanoparticle preparation, the aqueous plant extract was combined with 1 mM silver nitrate (AgNO_3_) solution at a ratio of 1:9 (v/v) while continuously stirred under ambient laboratory conditions. The initiation of nanoparticle formation was visually indicated by the progressive transformation of the reaction mixture into a dark brown color, reflecting the reduction of Ag^+^ ions and formation of nanosilver particles.

The reaction suspension was allowed to proceed for 24 h to promote complete bioreduction of silver ions. Following synthesis, the generated nanoparticles were isolated by centrifugation at 12,000 rpm for 20 min. The collected nanoparticle pellets were repeatedly rinsed with distilled water to remove residual plant-derived compounds and unreacted materials. Finally, the purified AgNPs were dried at 60 °C and preserved for subsequent physicochemical characterization analyses (Singh et al., 2018; Khan et al., 2018; Saravanan et al., 2021).

### UV–Vis spectroscopy

The optical properties and surface plasmon resonance (SPR) characteristics of the synthesized AgNPs were analyzed using a UV–visible spectrophotometer operating within a wavelength range of 200–900 nm (Shimadzu UV-1800, Japan). Prior to analysis, samples were appropriately diluted to avoid signal saturation. The obtained SPR absorption peaks were utilized to confirm nanoparticle synthesis and compare the optical behavior of nanoparticles synthesized from Syrian and Yemeni plant extracts (Vogt, Wondergem & Weckhuysen, 2023).

### Zeta potential analysis

The electrokinetic surface properties and dispersion stability of the synthesized nanoparticles were investigated using a Zetasizer instrument operating on electrophoretic mobility principles. Zeta potential values were estimated according to the Smoluchowski model to evaluate nanoparticle surface charge behavior and colloidal stability. To improve analytical consistency, all measurements were conducted in triplicate under identical experimental conditions (Hunter, 1981; Das et al., 2023).

### Scanning electron microscopy

Surface morphology and structural features of AgNPs synthesized from Syrian and Yemeni plant sources were characterized using scanning electron microscopy (JEOL JSM-IT500). Prior to imaging, dried nanoparticle powders were immobilized on carbon-supported aluminum stubs and coated with a thin conductive gold film to enhance image quality and electrical conductivity. The resulting scanning electron microscopy (SEM) images were examined to assess nanoparticle shape, surface appearance, particle distribution, and aggregation characteristics (Rigopoulos et al., 2024; Mustapha et al., 2022).

### Transmission electron microscopy (TEM)

Detailed evaluation of nanoparticle morphology and dimensional distribution was performed using transmission electron microscopy (TEM). A diluted suspension of the synthesized nanoparticles was carefully placed onto carbon-coated copper grids and left to dry under ambient conditions before microscopic examination. TEM imaging was carried out using a JEOL JEM-2100 instrument operated at an accelerating voltage of 200 kV. The obtained micrographs were utilized to determine nanoparticle morphology and estimate particle size distribution patterns.

### Energy-dispersive X-ray spectroscopy

Elemental analysis of the synthesized nanoparticles was performed using an energy-dispersive X-ray spectroscopy (EDS) detector integrated with the SEM system. The generated elemental spectra were examined to verify the presence of silver as the dominant constituent of the synthesized nanostructures. In addition, the analysis was used to detect other elements associated with residual phytochemical compounds originating from the plant extracts, which may contribute to nanoparticle capping and stabilization processes during biosynthesis (Rigopoulos et al., 2024).

### X-ray diffraction analysis

The structural crystallinity of the synthesized silver nanoparticles was investigated using X-ray diffraction (XRD) analysis. Diffraction profiles were collected using an X-ray diffractometer operated with Cu K*α* radiation (*λ* = 1.5406 Å) at 40 kV and 30 mA. Measurements were acquired within a 2*θ* scanning range of 15°–80° under optimized operating conditions to evaluate the crystalline structure, phase composition, and structural characteristics of the synthesized nanoparticles.

### Statistical analysis

Quantitative particle size analysis was performed using ImageJ software based on measurements of 50 randomly selected nanoparticles from each sample. Particle size data are presented as mean ± standard deviation (SD), together with particle size ranges and frequency distribution histograms. Physicochemical characterization results obtained from TEM, DLS, zeta potential, and XRD analyses were used for descriptive comparison of the nanoparticles synthesized from Syrian and Yemeni *T. vulgaris* extracts.

## Results

### UV–Vis spectroscopic analysis of AgNPs

**Figure 1:**
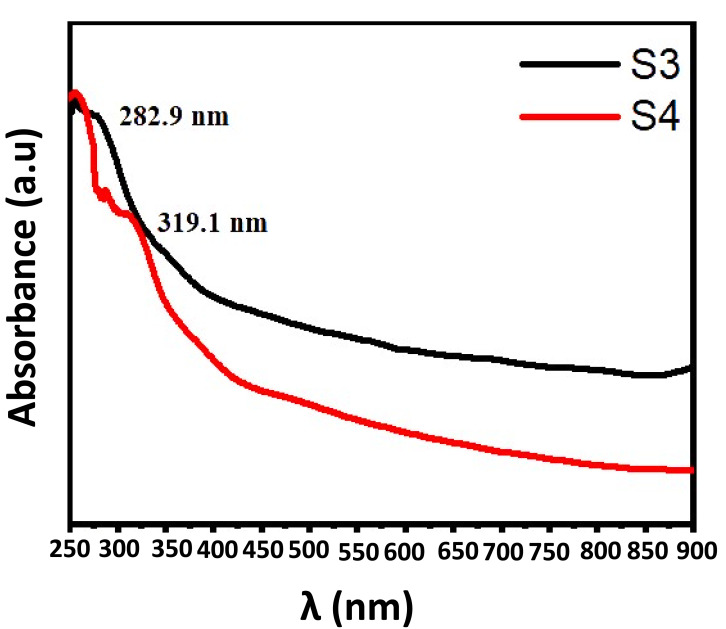
UV–Vis absorption spectra of reaction products obtained using aqueous extracts of *Thymus vulgaris* from Syrian and Yemeni commercial sources, showing absorption bands at 319.1 nm and 282.9 nm, respectively.

The optical properties of the reaction products obtained using *T. vulgaris* extracts from Yemeni and Syrian commercial sources were evaluated by UV–Vis spectroscopy over the wavelength range of 250–900 nm. The recorded spectra exhibited absorption bands at 282.9 nm and 319.1 nm for the Yemeni- and Syrian-derived samples, respectively (Fig. 1).

Differences in the absorption profiles between the two samples may reflect variations in particle characteristics, aggregation behavior, and interactions between silver species and plant-derived metabolites. The Yemeni-derived sample exhibited an absorption maximum at a lower wavelength than the Syrian-derived sample, suggesting differences in the physicochemical properties of the resulting products.

Because the observed absorption bands were located outside the typical surface plasmon resonance region commonly reported for silver nanoparticles, UV–Vis spectroscopy alone was not considered sufficient to confirm AgNP formation. Therefore, confirmation of nanoparticle synthesis was based on the combined evidence obtained from TEM, EDS, XRD, and zeta potential analyses. The UV–Vis results are presented primarily as complementary spectral information describing differences between the two synthesized samples.

### Zeta potential analysis

**Figure 2:**
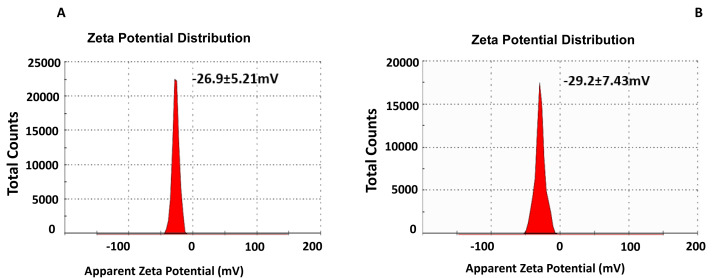
Zeta potential distribution of silver nanoparticles synthesized using *Thymus vulgaris* extracts: (A) Syrian-derived AgNPs exhibiting a zeta potential of −26. 9 mV and (B) Yemeni-derived AgNPs exhibiting a zeta potential of −30.1 mV, indicating negatively charged nanoparticle surfaces and moderate colloidal stability.

Zeta potential measurements were performed to evaluate the surface charge characteristics and colloidal stability of the synthesized silver nanoparticles. As shown in Fig. 2, both nanoparticle samples exhibited negative zeta potential values, indicating the presence of negatively charged species on the nanoparticle surfaces. The Syrian-derived AgNPs (Fig. 2A) showed a zeta potential value of −26.9 mV, whereas the Yemeni-derived AgNPs (Fig. 2B) exhibited a slightly more negative value of −30.1 mV.

 Comparable zeta potential distribution profiles were observed for both nanoparticle preparations, suggesting similar surface charge characteristics and stabilization mechanisms. The negative surface charge may be attributed to the adsorption of plant-derived biomolecules, including phenolic and flavonoid compounds, onto the nanoparticle surface during the biosynthesis process. Such surface-associated compounds can contribute to nanoparticle stabilization by generating electrostatic repulsion between particles in suspension.

The observed zeta potential values suggest moderate colloidal stability for both nanoparticle preparations. The slightly higher negative charge observed for the Yemeni-derived AgNPs may indicate improved electrostatic stabilization compared with the Syrian-derived sample, although both preparations remained within a comparable stability range.

### Scanning electron microscopy analysis

**Figure 3:**
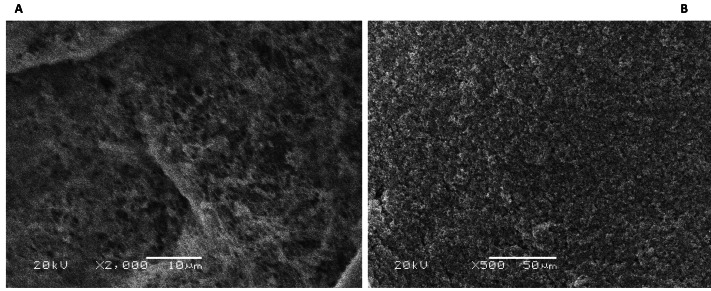
Scanning electron microscopy (SEM) images of silver nanoparticles synthesized using *Thymus vulgaris* extracts from different geographical origins: (A) Syrian sample and (B) Yemeni sample showing aggregated nanoscale structures formed during the green synthesis process.

The surface architecture and morphological features of the biosynthesized silver nanoparticles were examined through SEM. As presented in Fig. 3, AgNPs synthesized using *T. vulgaris* extracts from Syrian and Yemeni origins displayed nanoscale particulate structures with noticeable aggregation behavior. The Syrian-derived nanoparticles (Fig. 3A) formed compact irregular assemblies distributed across the sample surface, indicating the presence of interconnected nanoparticle networks. Similarly, the Yemeni-derived nanoparticles (Fig. 3B) also exhibited clustered nanosized structures with varying aggregation patterns.

 Such aggregation phenomena are frequently observed in biologically synthesized nanoparticles and are commonly linked to interactions between nanoparticle surfaces and residual phytochemical constituents that function as natural stabilizing and capping agents. Nevertheless, the SEM micrographs clearly confirmed the successful generation of silver nanoparticles through the plant-mediated green synthesis route.

### Transmission electron microscopy analysis of syrian-derived AgNPs

**Figure 4:**
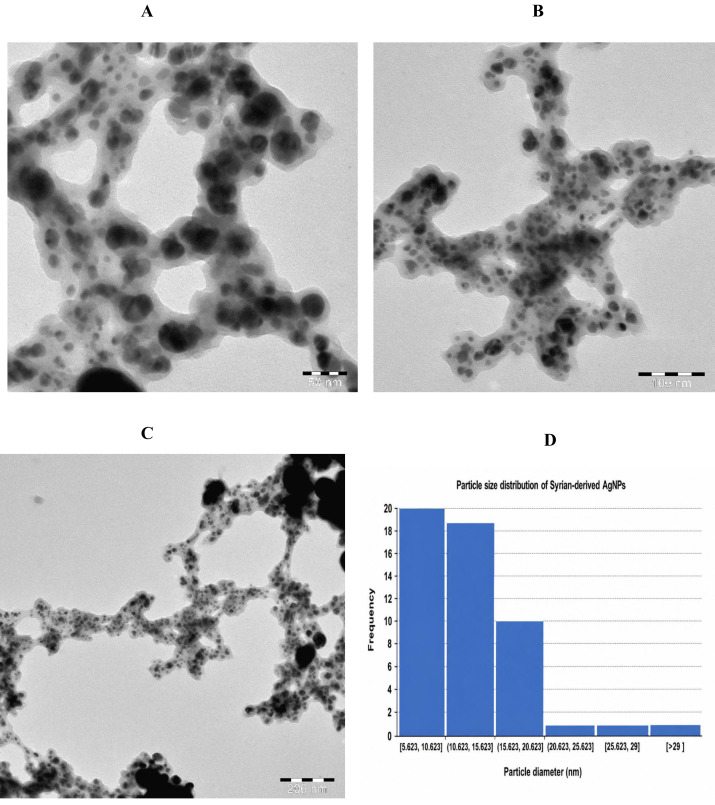
Transmission electron microscopy (TEM) images of silver nanoparticles synthesized using Syrian *Thymus vulgaris* extract at different magnifications (A–C). (D) Particle size distribution histogram generated from ImageJ measurements of 50 randomly selected nanoparticles. The average particle diameter was 13.38 ± 5.26 nm, with particle sizes ranging from 5.62 to 33.21 nm.

Detailed evaluation of the morphology and size distribution of silver nanoparticles synthesized using Syrian *Thymus vulgaris* extract was performed by TEM. As shown in Fig. 4, the synthesized nanoparticles were predominantly spherical in shape and exhibited varying degrees of aggregation, forming interconnected nanoscale assemblies.

TEM micrographs obtained at different magnifications revealed that the nanoparticles were embedded within a network-like matrix, which may be attributed to the adsorption of plant-derived biomolecules acting as natural capping and stabilizing agents during nanoparticle formation. Quantitative particle size analysis was conducted using ImageJ software based on measurements of 50 randomly selected nanoparticles. The average particle diameter was 13.38 ± 5.26 nm, with particle sizes ranging from 5.62 to 33.21 nm (Fig. 4D). The particle size distribution histogram indicated that the majority of nanoparticles were distributed within the 5–20 nm range.

The clustering behavior observed in the TEM micrographs is commonly associated with biosynthesized nanoparticles and may result from interactions between nanoparticle surfaces and residual phytochemical constituents originating from the plant extract. Despite partial agglomeration, individual nanoparticles remained distinguishable and retained their nanoscale morphology, confirming the successful green synthesis of silver nanoparticles.

### Transmission electron microscopy analysis of Yemeni-derived AgNPs

**Figure 5:**
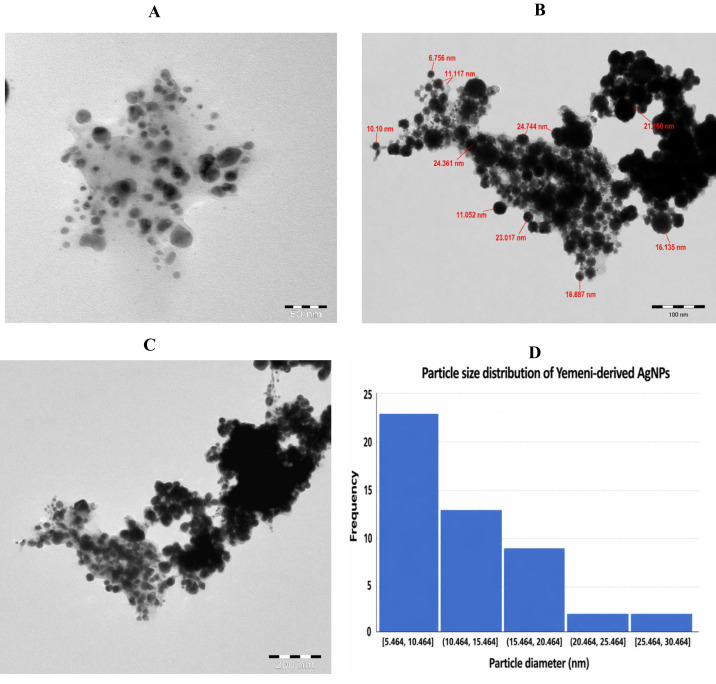
Transmission electron microscopy (TEM) images of silver nanoparticles synthesized using Yemeni *Thymus vulgaris* extract at different magnifications (A–C). (D) Particle size distribution histogram generated from ImageJ measurements of 50 randomly selected nanoparticles. The average particle diameter was 12.34 ± 5.31 nm, with particle sizes ranging from 5.46 to 26.72 nm.

The morphological characteristics and particle size distribution of silver nanoparticles synthesized using Yemeni *Thymus vulgaris* extract were investigated using TEM. Representative micrographs obtained at different magnifications (Fig. 5) revealed predominantly spherical nanoparticles with varying degrees of aggregation, forming interconnected nanoscale clusters.

Discrete nanoparticles were clearly distinguishable within the aggregated network, suggesting successful nanoparticle formation and stabilization by plant-derived biomolecules. Quantitative particle size analysis was performed using ImageJ software based on measurements of 50 randomly selected nanoparticles. The average particle diameter was 12.34 ± 5.31 nm, with particle sizes ranging from 5.46 to 26.72 nm (Fig. 5D). The particle size distribution histogram demonstrated that most nanoparticles were distributed within the 5–20 nm size range.

The aggregation observed in the TEM micrographs may be attributed to interactions between nanoparticle surfaces and residual phytochemical constituents present in the plant extract, which act as reducing, capping, and stabilizing agents during biosynthesis. Despite partial agglomeration, individual nanoparticles remained clearly identifiable and retained their nanoscale morphology, confirming the successful green synthesis of silver nanoparticles and controlled particle growth mediated by plant-derived metabolites.

Quantitative TEM analysis revealed comparable nanoparticle dimensions for both preparations. Syrian-derived AgNPs exhibited an average particle diameter of 13.38 ± 5.26 nm, whereas Yemeni-derived AgNPs showed an average diameter of 12.34 ± 5.31 nm. Particle size distribution histograms obtained from ImageJ measurements of 50 randomly selected nanoparticles are presented in Figs. 4D and 5D. The results indicate that both extracts produced predominantly small spherical nanoparticles within a similar nanoscale range.

### Elemental composition analysis

**Figure 6:**
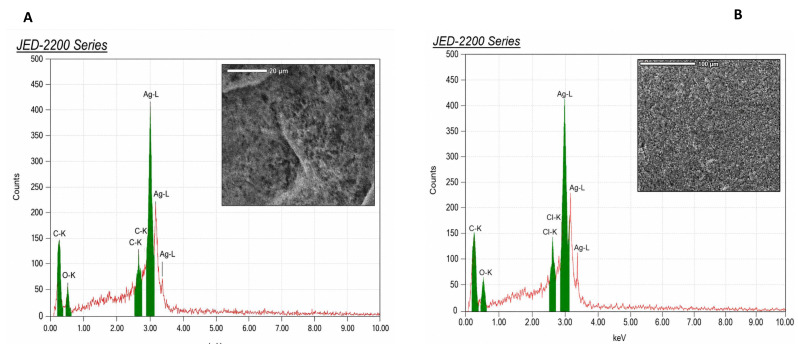
Energy-dispersive X-ray spectroscopy (EDS) spectra accompanied by SEM micrographs of silver nanoparticles synthesized using Manuscript extracts from two geographical origins: (A) Syrian source and (B) Yemeni source. Both spectra revealed a dominant silver peak near 3 keV, confirming the successful synthesis of AgNPs, whereas smaller carbon and oxygen signals were attributed to phytochemical residues associated with nanoparticle surface stabilization.

Elemental characterization of the biosynthesized silver nanoparticles was performed using energy-dispersive X-ray spectroscopy (EDS). The spectra presented in Fig. 6 demonstrated that nanoparticles synthesized from Syrian (Fig. 6A) and Yemeni (Fig. 6B) *Thymus vulgaris* extracts contained a pronounced silver signal around 3 keV, which is characteristic of metallic silver and confirms its predominance within the synthesized nanostructures.

Quantitative EDS analysis further confirmed silver as the major elemental constituent of both nanoparticle preparations. The detailed elemental composition, expressed as weight percentage (wt%) and atomic percentage (at%), is presented in Table 1. Silver represented the dominant element in both samples, while lower amounts of carbon, oxygen, and chlorine were also detected.

The detected carbon and oxygen signals are likely associated with residual plant-derived organic compounds adsorbed onto nanoparticle surfaces following biosynthesis. Such surface-associated biomolecules may contribute to nanoparticle stabilization by acting as naturally occurring capping agents. Furthermore, the inset SEM images included in Fig. 6 provided additional visualization of nanoparticle morphology and surface distribution characteristics.

### X-ray diffraction analysis

**Figure 7:**
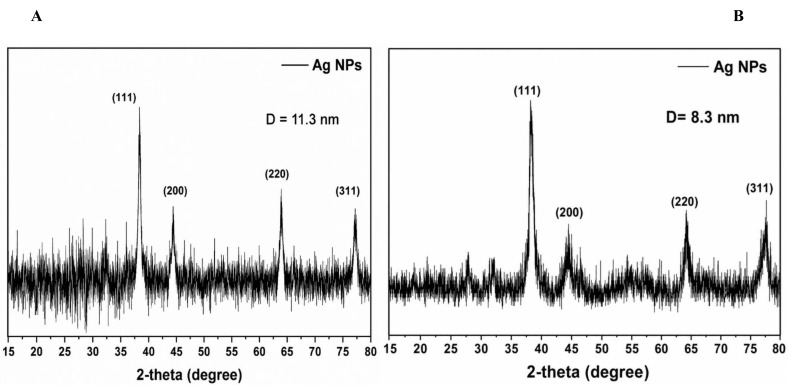
X-ray diffraction (XRD) patterns of silver nanoparticles synthesized using *Thymus vulgaris* extracts from different geographical origins: (A) Syrian-derived AgNPs and (B) Yemeni-derived AgNPs. Diffraction peaks indexed to the (111), (200), (220), and (311) planes confirmed the face-centered cubic (fcc) crystalline structure of metallic silver. The average crystallite sizes calculated using the Scherrer equation were 11.3 nm and 8.3 nm for Syrian- and Yemeni-derived AgNPs, respectively.

The crystalline structure of the biosynthesized silver nanoparticles was investigated using X-ray diffraction (XRD) analysis. As shown in Fig. 7, AgNPs synthesized using Syrian (Fig. 7A) and Yemeni (Fig. 7B) *T. vulgaris* extracts exhibited characteristic diffraction peaks at approximately 2*θ* values of 38°, 44°, 64°, and 77°, corresponding to the (111), (200), (220), and (311) crystallographic planes of metallic silver, respectively.

**Table 1:** Elemental composition of AgNPs determined by EDS analysis.

**Syrian AgNPs (wt%)**	**Syrian AgNPs (at%)**	**Yemeni AgNPs (wt%)**	**Yemeni AgNPs (at%)**	**Element**
6.45	26.38	10.73	34.92	C
11.36	34.90	14.04	34.28	O
1.38	1.91	4.80	5.29	Cl
80.81	36.81	70.42	25.51	Ag

**Notes.**

EDS energy-dispersive X-ray spectroscopy wt% weight percentage at% atomic percentage

The diffraction pattern confirmed the formation of crystalline silver nanoparticles with a face-centered cubic (fcc) structure. Among the detected reflections, the (111) plane exhibited the highest intensity, indicating preferential crystal orientation along this plane. The sharp and well-defined diffraction peaks further demonstrated the high crystallinity of the synthesized nanoparticles. The average crystallite size was estimated using the Scherrer equation and was found to be approximately 11.3 nm for the Syrian-derived AgNPs and 8.3 nm for the Yemeni-derived AgNPs. These values are consistent with the nanoscale particle dimensions observed by TEM analysis and further confirm the successful formation of crystalline silver nanoparticles.

Furthermore, the absence of additional diffraction peaks associated with secondary crystalline phases indicated a relatively high degree of phase purity. Minor background signals observed in some regions of the diffractograms may be attributed to trace organic residues originating from plant-derived compounds adsorbed onto the nanoparticle surfaces during the biosynthesis process.

## Discussion

Our study demonstrated the successful green synthesis of silver nanoparticles (AgNPs) using aqueous extracts of Syrian and Yemeni *T. vulgaris*. The formation of AgNPs was verified through a combination of physicochemical characterization techniques, including UV–Vis spectroscopy, dynamic light scattering (DLS), zeta potential analysis, SEM, TEM, EDS, and XRD. Collectively, these analyses confirmed the production of nanoscale crystalline silver particles through an environmentally friendly biosynthetic approach. Plant-mediated synthesis has been widely recognized as a sustainable alternative to conventional chemical methods because plant-derived metabolites can participate in both the reduction of metal ions and stabilization of the resulting nanoparticles (Ahmed et al., 2016; Bao et al., 2021; Wahab et al., 2021).

UV–Vis spectroscopy revealed distinct absorption profiles for the Syrian- and Yemeni-derived nanoparticle preparations. Although the absorption behavior differed between the two preparations, UV–Vis spectroscopy alone was not considered sufficient to confirm nanoparticle formation. Instead, nanoparticle formation was supported by complementary characterization techniques, including DLS, TEM, EDS, and XRD analyses. The observed spectral differences may be associated with variations in nanoparticle size distribution, aggregation state, surface characteristics, or the nature of biomolecules adsorbed onto nanoparticle surfaces. Consistent with this observation, DLS analysis revealed differences in hydrodynamic particle size between the two nanoparticle preparations. However, because detailed phytochemical characterization was not performed in the present study, the specific factors responsible for the observed spectral differences cannot be conclusively identified (Vogt, Wondergem & Weckhuysen, 2023).

Particle size characterization revealed notable differences between DLS and TEM measurements. DLS analysis indicated average hydrodynamic diameters of approximately 84.18 nm for Syrian-derived AgNPs and 125.2 nm for Yemeni-derived AgNPs, whereas quantitative TEM analysis based on measurements of 50 randomly selected nanoparticles yielded average particle diameters of 13.38 ± 5.26 nm and 12.34 ± 5.31 nm, respectively. Such discrepancies are commonly observed because DLS measures the hydrodynamic diameter of particles suspended in solution, including associated solvent layers, adsorbed biomolecules, and particle aggregates, whereas TEM measures the physical dimensions of individual nanoparticle cores after sample preparation and drying (Das et al., 2023). The larger hydrodynamic sizes observed by DLS therefore likely reflect aggregation phenomena and the presence of phytochemical constituents adsorbed onto nanoparticle surfaces.

The particle size distribution histograms obtained from TEM measurements demonstrated that most nanoparticles were distributed within the 5–20 nm range for both preparations. Furthermore, the relatively similar mean particle sizes obtained for Syrian- and Yemeni-derived AgNPs suggest that both T. vulgaris extracts were comparably effective in mediating silver ion reduction and nanoparticle formation under the experimental conditions employed. our results are generally consistent with previous reports describing the successful biosynthesis of AgNPs using *Thymus vulgaris* and other Thymus species (Ejaz et al., 2024; Şimşek, 2025). Zeta potential measurements revealed negative surface charges of −26.9 mV and −30.1 mV for Syrian- and Yemeni-derived nanoparticles, respectively. Negative zeta potential values are commonly associated with the adsorption of negatively charged biomolecules onto nanoparticle surfaces and may contribute to electrostatic repulsion between particles, thereby reducing aggregation tendencies in suspension (Hunter, 1981; Das et al., 2023). The slightly more negative value observed for the Yemeni-derived nanoparticles may indicate somewhat greater electrostatic stabilization. However, because long-term colloidal stability was not evaluated in our study, definitive conclusions regarding storage stability cannot be drawn. SEM and TEM analyses demonstrated that the synthesized nanoparticles were predominantly spherical and exhibited varying degrees of aggregation. Similar clustering behavior has been reported frequently in biosynthesized nanoparticle systems and is often attributed to interactions among nanoparticles and residual surface-bound biomolecules derived from plant extracts (Balciunaitiene et al., 2021; Rigopoulos et al., 2024). Despite the presence of localized aggregates, individual nanoparticles remained clearly distinguishable, confirming successful nanoparticle formation at the nanoscale level. Elemental composition analysis by EDS confirmed silver as the predominant constituent of the synthesized nanoparticles, supporting the successful reduction of silver ions during the biosynthesis process. In addition, XRD analysis revealed characteristic diffraction peaks at approximately 38°, 44°, 64°, and 77°, corresponding to the (111), (200), (220), and (311) crystallographic planes of metallic silver. These reflections confirmed the formation of crystalline silver nanoparticles with a face-centered cubic (fcc) structure, which is consistent with previously reported plant-mediated AgNP systems (Balciunaitiene et al., 2021; Rigopoulos et al., 2024). Crystallite sizes calculated using the Scherrer equation were approximately 11.3 nm for Syrian-derived AgNPs and 8.3 nm for Yemeni-derived AgNPs. These values were slightly smaller than the corresponding particle sizes obtained by TEM analysis, which is expected because XRD estimates the size of coherent crystalline domains whereas TEM measures the overall particle dimensions. The relatively close agreement between TEM-derived particle sizes and XRD-derived crystallite sizes further supports the successful synthesis of highly crystalline nanoscale silver particles.

The face-centered cubic crystalline structure identified in our investigation is also in agreement with previous studies of thyme-mediated AgNPs, which consistently reported characteristic diffraction reflections corresponding to metallic silver and confirmed the formation of crystalline nanostructures (Ejaz et al., 2024; Şimşek, 2025).

Although certain physicochemical differences were observed between the two nanoparticle preparations, our study was not designed to determine the specific phytochemical factors responsible for these variations. Previous studies have reported considerable chemical variability among Thymus species and populations, including differences in phenolic compounds, terpenoids, and essential oil composition (Sarfaraz, Rahimmalek & Saeidi, 2021; Etri & Pluhár, 2024; Zinno et al., 2023). Nevertheless, no phytochemical characterization was performed in the current investigation; therefore, any association between nanoparticle properties and phytochemical composition should be considered preliminary and requires further experimental verification. Overall, the results demonstrate that Syrian and Yemeni *T. vulgaris* extracts can serve as effective reducing and stabilizing agents for the green synthesis of crystalline silver nanoparticles. Future investigations should incorporate detailed phytochemical profiling, long-term stability studies, and biological activity assessments to better elucidate the mechanisms governing nanoparticle formation and functional performance.

The average particle diameters obtained in the present study (13.38 ± 5.26 nm and 12.34 ± 5.31 nm) are generally comparable to those reported for thyme-mediated AgNPs in previous investigations. Ejaz et al. (2024) similarly reported the successful synthesis of nanoscale silver particles using *T. vulgaris* extracts, whereas Şimşek (2025) demonstrated that Thymus species can effectively mediate the formation of crystalline AgNPs with characteristic spherical morphology. The predominance of spherical nanoparticles observed in our study is therefore consistent with previous reports involving thyme-assisted nanoparticle biosynthesis.

### Study limitations

Our study has several limitations that should be considered when interpreting the findings. First, detailed phytochemical characterization of the *T. vulgaris* extracts was not performed; therefore, direct relationships between extract composition and nanoparticle properties could not be established. Second, although zeta potential measurements provided information regarding nanoparticle surface charge characteristics, long-term colloidal stability was not evaluated. Third, the investigation focused primarily on physicochemical characterization and did not include biological activity assessments such as antimicrobial, antioxidant, or cytotoxicity testing. Finally, the comparison was limited to two commercial thyme sources, and broader sampling may provide additional insight into source-dependent variability. Future studies incorporating comprehensive phytochemical profiling, stability assessments, and biological evaluations would further enhance understanding of thyme-mediated silver nanoparticle synthesis and functionality.

## Conclusions

The present study demonstrated the successful green synthesis of silver nanoparticles (AgNPs) using aqueous extracts of *Thymus vulgaris* through an environmentally friendly approach. Comprehensive physicochemical characterization confirmed nanoparticle formation and provided detailed information regarding their structural, morphological, and surface properties. UV–Vis spectroscopy revealed characteristic absorption profiles associated with nanoparticle formation, while DLS and zeta potential analyses provided information regarding hydrodynamic particle size distribution and surface charge characteristics. SEM and TEM analyses demonstrated the formation of predominantly spherical nanoparticles with nanoscale dimensions, whereas quantitative TEM measurements confirmed average particle diameters of 13.38 ± 5.26 nm and 12.34 ± 5.31 nm for Syrian- and Yemeni-derived AgNPs, respectively. EDS analysis verified silver as the major elemental constituent, and XRD analysis confirmed the crystalline metallic nature of the nanoparticles with a characteristic face-centered cubic (fcc) structure.

Overall, the results indicate that *Thymus vulgaris* extracts can serve as effective reducing and stabilizing agents for the biosynthesis of crystalline silver nanoparticles. Although minor physicochemical differences were observed between the two nanoparticle preparations, the present study was not designed to determine the specific phytochemical factors responsible for these variations. Future studies incorporating detailed phytochemical profiling, long-term stability assessments, and biological activity evaluations are warranted to further elucidate the mechanisms governing nanoparticle formation and functional performance. In addition, the physicochemical characteristics observed in the synthesized AgNPs provide a basis for future investigations exploring their potential applications in antimicrobial materials, nanobiotechnology, and other biomedical and technological fields. However, such applications require further biological and functional evaluation before practical implementation can be considered.

##  Supplemental Information

10.7717/peerj.21583/supp-1 Supplemental Information 1 Raw UV–Vis spectral data of silver nanoparticles synthesized using *Thymus vulgaris* extracts from Syrian and Yemeni origins Raw UV–Vis spectral data for silver nanoparticles synthesized using *Thymus vulgaris* extracts (Syrian and Yemeni samples), including wavelength (nm) and absorbance values used to confirm nanoparticle formation.

10.7717/peerj.21583/supp-2 Supplemental Information 2 Zeta potential measurements of silver nanoparticles synthesized using plant extracts Raw zeta potential data for silver nanoparticles synthesized using *Thymus vulgaris* extracts (Syrian samples), including measured surface charge values used to assess colloidal stability.

10.7717/peerj.21583/supp-3 Supplemental Information 3 Zeta potential measurements of silver nanoparticles synthesized using plant extracts Raw zeta potential data for silver nanoparticles synthesized using *Thymus vulgaris* extracts (Yemeni samples), including measured surface charge values used to assess colloidal stability.

10.7717/peerj.21583/supp-4 Supplemental Information 4 Energy-dispersive X-ray spectroscopy (EDS) data showing elemental composition of silver nanoparticles synthesized using *Thymus vulgaris* extracts

10.7717/peerj.21583/supp-5 Supplemental Information 5 Energy-dispersive X-ray spectroscopy (EDS) data showing elemental composition of silver nanoparticles synthesized using *Thymus vulgaris* extracts Raw EDS data for silver nanoparticles (AgNPs) synthesized using *Thymus vulgaris* extracts (Syrian and Yemeni samples), including elemental spectra confirming the presence of silver (Ag).

## Abbreviations

AgNPs: Silver nanoparticles
DLS: Dynamic light scattering
EDS: Energy-dispersive X-ray spectroscopy
SEM: Scanning electron microscopy
TEM: Transmission electron microscopy
UV–Vis: Ultraviolet–visible spectroscopy
XRD: X-ray diffraction
SPR: Surface plasmon resonance
PDI: Polydispersity index

## Additional Information and Declarations

### Data Availability

The following information was supplied regarding data availability: The raw data is available in the Supplemental Files.

## References

[ref-1] Aboyewa JA, Sibuyi NR, Meyer M, Oguntibeju OO (2021). Green synthesis of metallic nanoparticles using selected medicinal plants from southern Africa and their biological applications. Plants.

[ref-2] Ahmed S, Ahmad M, Swami BL, Ikram S (2016). Green synthesis of silver nanoparticles using Azadirachta indica aqueous leaf extract. Journal of Radiation Research and Applied Sciences.

[ref-3] Aldosary SK, El-Rahman SNA, Al-Jameel SS, Alromihi NM (2021). Antioxidant and antimicrobial activities of *Thymus vulgaris* essential oil and synthesized silver nanoparticles. Brazilian Journal of Biology.

[ref-4] Balciunaitiene A, Viskelis P, Viskelis J, Streimikyte P, Liaudanskas M, Bartkiene E, Zavistanaviciute P, Zokaityte E, Starkute V, Ruzauskas M, Lele V (2021). Green synthesis of silver nanoparticles using extract of *Artemisia absinthium L.*, *Humulus lupulus L.* and *Thymus vulgaris L.*, physico-chemical characterization, antimicrobial and antioxidant activity. Processes.

[ref-5] Bao Y, He J, Song K, Guo J, Zhou X, Liu S (2021). Plant-extract-mediated synthesis of metal nanoparticles. Journal of Chemistry.

[ref-6] Bruna T, Maldonado-Bravo F, Jara P, Caro N (2021). Silver nanoparticles and their antibacterial applications. International Journal of Molecular Sciences.

[ref-7] Das SK, Chakraborty S, Rajabalaya R, Mazumder B (2023). Zeta potential measurements and analysis for biomedical nanotechnology. Analytical techniques for biomedical nanotechnology.

[ref-8] Dhir R, Chauhan S, Subham P, Kumar S, Sharma P, Shidiki A, Kumar G (2023). Plant-mediated synthesis of silver nanoparticles: unlocking their pharmacological potential—a comprehensive review. Frontiers in Bioengineering and Biotechnology.

[ref-9] Ejaz U, Afzal M, Mazhar M, Riaz M, Ahmed N, Rizg WY, Alahmadi AA, Badr M, Mushtaq RY, Yean CY (2024). Characterization, synthesis, and biological activities of silver nanoparticles produced *via* green synthesis method using *Thymus Vulgaris* aqueous extract. International Journal of Nanomedicine.

[ref-10] Etri K, Pluhár Z (2024). Exploring chemical variability in the essential oils of the Thymus genus. Plants.

[ref-11] Hunter RJ (1981). Zeta potential in colloid science: principles and applications.

[ref-12] Ilić Z, Stanojević L, Milenković L, Šunić L, Milenković A, Stanojević J, Cvetković D (2022). The yield, chemical composition, and antioxidant activities of essential oils from different plant parts of the wild and cultivated oregano (*Origanum vulgare L.*). Horticulturae.

[ref-13] Khalifa HO, Oreiby A, Mohammed T, Abdelhamid MA, Sholkamy EN, Hashem H, Fereig RM (2025). Silver nanoparticles as next-generation antimicrobial agents: mechanisms, challenges, and innovations against multidrug-resistant bacteria. Frontiers in Cellular and Infection Microbiology.

[ref-14] Khan M, Shaik MR, Adil SF, Khan ST, Al-Warthan A, Siddiqui MRH (2018). Plant extracts as green reductants for the synthesis of silver nanoparticles: Lessons from chemical synthesis. Dalton Transactions.

[ref-15] Lithi IJ, Nakib KIA, Chowdhury AS, Hossain MS (2025). A review on the green synthesis of metal (Ag, Cu, and Au) and metal oxide (ZnO, MgO, Co_3_O_4_, and TiO_2_) nanoparticles using plant extracts for developing antimicrobial properties. Nanoscale Advances.

[ref-16] Mustapha T, Misni N, Ithnin NR, Daskum AM, Unyah NZ (2022). A review on plants and microorganisms mediated synthesis of silver nanoparticles, role of plants metabolites and applications. International Journal of Environmental Research and Public Health.

[ref-17] Rigopoulos N, Gkaliouri CM, Ioannou Z, Giaouris E, Sakavitsi V, Gournis D (2024). Green synthesis of silver nanoparticles using plant waste extracts. Nano Express.

[ref-18] Saravanan A, Kumar PS, Karishma S, Vo DVN, Jeevanantham S, Yaashikaa PR, George CS (2021). Biosynthesis of metal nanoparticles and environmental applications. Chemosphere.

[ref-19] Sarfaraz D, Rahimmalek M, Saeidi G (2021). Polyphenolic and molecular variation in Thymus species. Scientific Reports.

[ref-20] Şimşek S (2025). Green synthesis and bioactivity of Thymus pectinatus-derived silver nanoparticles. ChemistrySelect.

[ref-21] Singh D, Kumar V, Yadav E, Falls N, Singh M, Komal U, Verma A (2018). One-pot green synthesis and structural characterisation of silver nanoparticles using aqueous leaves extract of Carissa carandas: antioxidant, anticancer and antibacterial activities. IET Nanobiotechnology.

[ref-22] Thatyana M, Dube NP, Kemboi D, Manicum ALE, Mokgalaka-Fleischmann NS, Tembu JV (2023). Advances in phytonanotechnology and plant-mediated nanoparticle synthesis. Nanomaterials.

[ref-23] Thomas S, Gonsalves RA, Jose J, Zyoud SH, Prasad AR, Garvasis J (2024). Plant-based synthesis, characterization approaches, applications and toxicity of silver nanoparticles: a comprehensive review. Journal of Biotechnology.

[ref-24] Vogt C, Wondergem CS, Weckhuysen BM (2023). Ultraviolet–visible (UV–Vis) spectroscopy. Springer handbook of advanced catalyst characterization.

[ref-25] Wahab S, Khan T, Adil M, Khan A (2021). Mechanistic aspects of plant-based silver nanoparticles. Heliyon.

[ref-26] Zinno P, Guantario B, Lombardi G, Ranaldi G, Finamore A, Allegra S, Mammano MM, Fascella G, Raffo A, Roselli M (2023). Chemical composition of essential oils in plant genotypes. Plants.

