# Supplementary material for: Comparative green synthesis and physicochemical characterization of silver nanoparticles using Thymus vulgaris extracts from Syrian and Yemeni sources
Source: PeerJ. 2026 Aug 4;14:e21583. doi: 10.7717/peerj.21583 (PMC13450036; doi:10.7717/peerj.21583)

# Zeta Potential Report

*v2.3*

Malvern Instruments Ltd - © Copyright 2008

## Sample Details

**Sample Name:** S3-Ay 1

**SOP Name:** mansettings.nano

**General Notes:**

| | | | |
|---|---|---|---|
| **File Name:** | Zeta 2.dts | **Dispersant Name:** | Water |
| **Record Number:** | 249 | **Dispersant RI:** | 1.330 |
| **Date and Time:** | Monday, September 09, 2013 5:3... | **Viscosity (cP):** | 0.8872 |
| | | **Dispersant Dielectric Constant:** | 78.5 |

## System

| | | | |
|---|---|---|---|
| **Temperature (°C):** | 25.1 | **Zeta Runs:** | 12 |
| **Count Rate (kcps):** | 80.7 | **Measurement Position (mm):** | 2.00 |
| **Cell Description:** | Clear disposable zeta cell | **Attenuator:** | 8 |

## Results

| | | | Mean (mV) | Area (%) | St Dev (mV) |
|---|---|---|---|---|---|
| **Zeta Potential (mV):** | -26.9 | **Peak 1:** | -26.9 | 100.0 | 5.21 |
| **Zeta Deviation (mV):** | 5.21 | **Peak 2:** | 0.00 | 0.0 | 0.00 |
| **Conductivity (mS/cm):** | 0.773 | **Peak 3:** | 0.00 | 0.0 | 0.00 |

**Result quality :** **See result quality report**

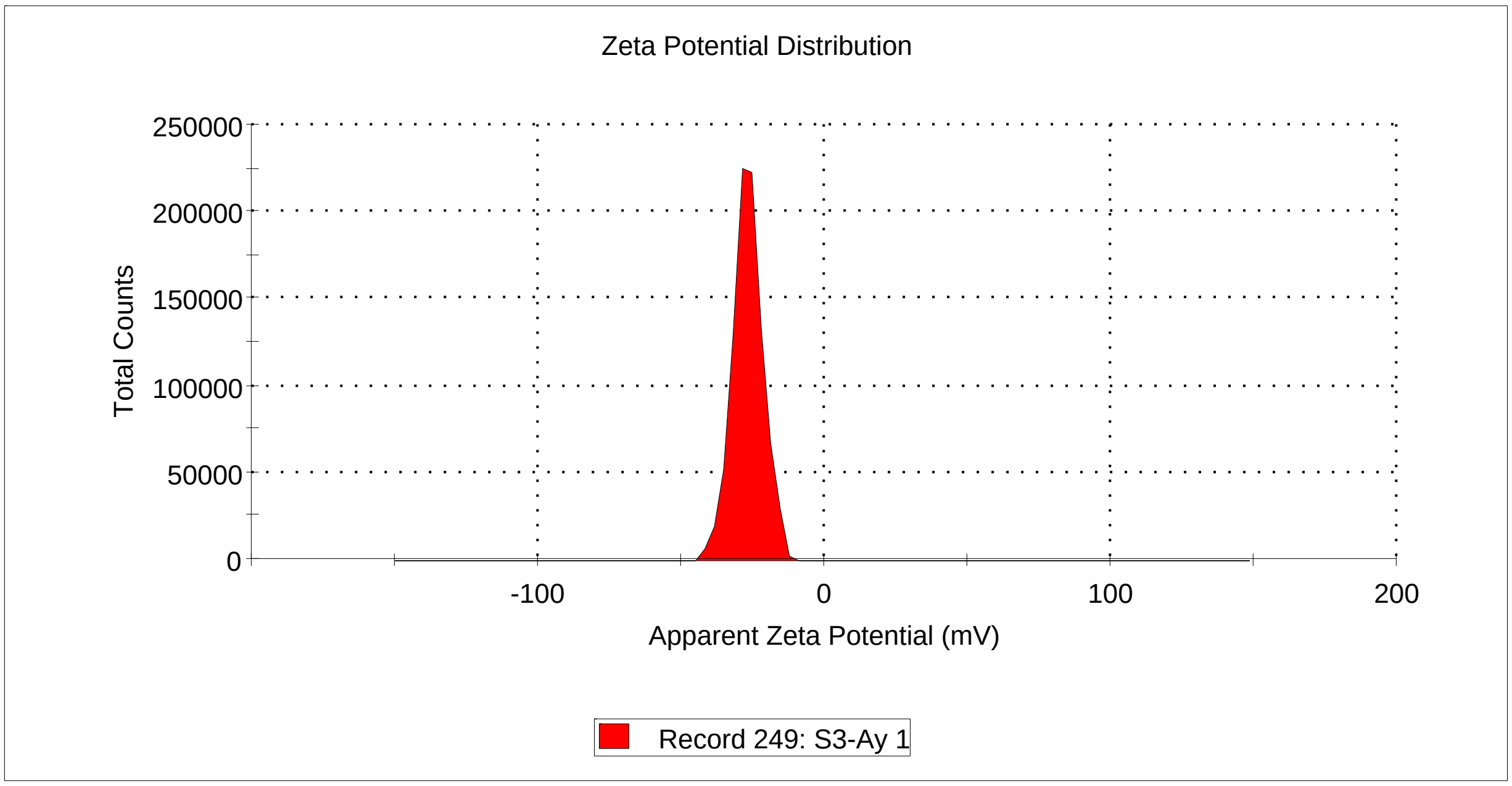

Supplement: Supplementary file 1 [file peerj-14-21583-s002.pdf]
