# Supplementary material for: Comparative green synthesis and physicochemical characterization of silver nanoparticles using Thymus vulgaris extracts from Syrian and Yemeni sources
Source: PeerJ. 2026 Aug 4;14:e21583. doi: 10.7717/peerj.21583 (PMC13450036; doi:10.7717/peerj.21583)

Zeta Potential Report

v2.3

Malvern Instruments Ltd - © Copyright 2008

Sample Details

Sample Name: S4-Ay 3

SOP Name: mansettings.nano

General Notes:

**File Name:**	Zeta 2.dts	**Dispersant Name:**	Water
**Record Number:**	254	**Dispersant RI:**	1.330
**Date and Time:**	Monday, September 09, 2013 5:4...	**Viscosity (cP):**	0.8872
		**Dispersant Dielectric Constant:**	78.5

System

**Temperature (°C):**	24.9	**Zeta Runs:**	12
**Count Rate (kcps):**	161.7	**Measurement Position (mm):**	2.00
**Cell Description:**	Clear disposable zeta cell	**Attenuator:**	8

Results

			Mean (mV)	Area (%)	St Dev (mV)
**Zeta Potential (mV):**	-30.1	**Peak 1:**	-29.2	100.0	7.43
**Zeta Deviation (mV):**	14.0	**Peak 2:**	0.00	0.0	0.00
**Conductivity (mS/cm):**	0.765	**Peak 3:**	0.00	0.0	0.00

Result quality : **See result quality report**

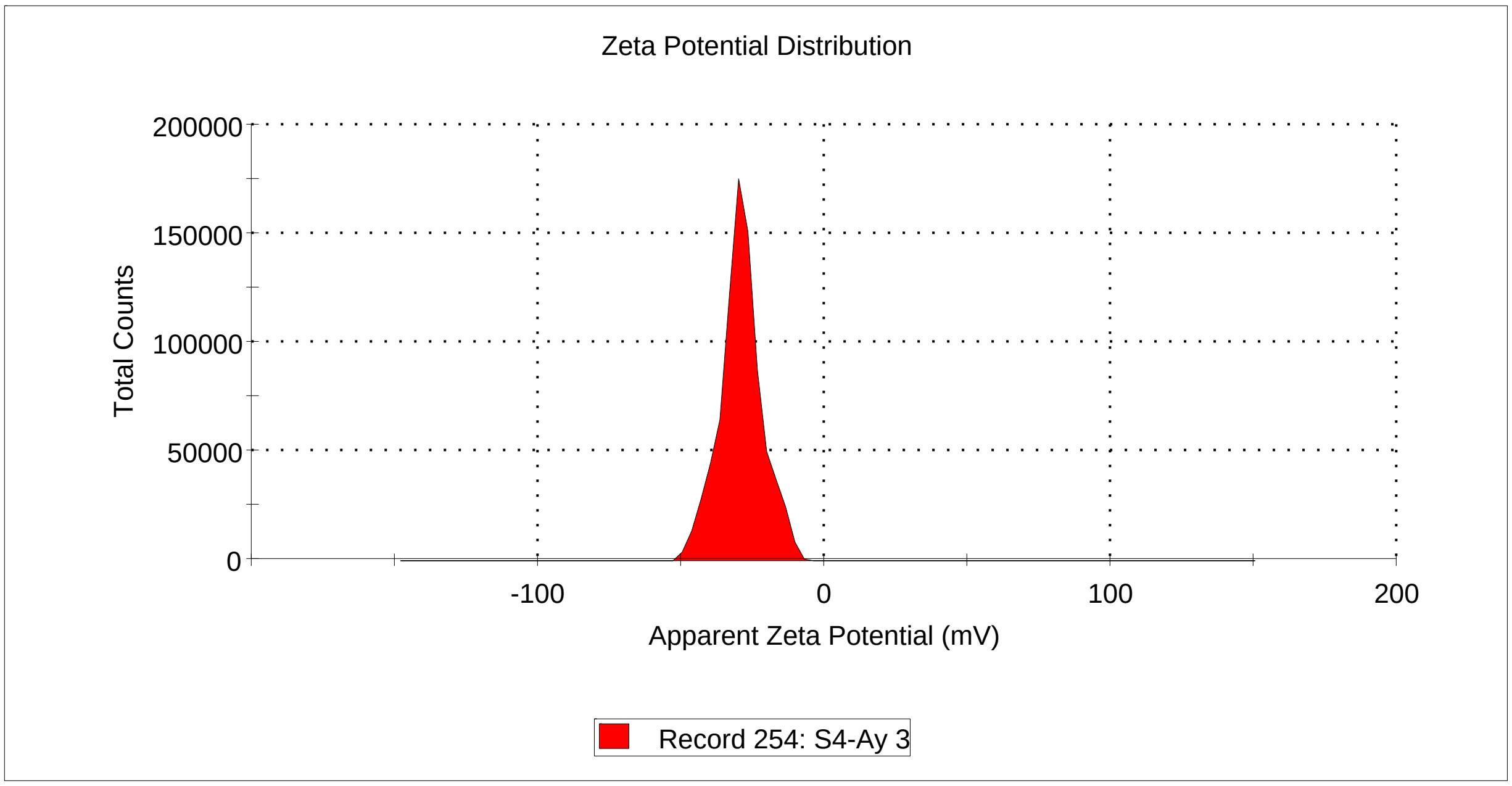

Supplement: Supplementary file 2 [file peerj-14-21583-s003.pdf]
